# Design of a Dyadic Digital Health Module for Chronic Disease Shared Care: Development Study

**DOI:** 10.2196/45035

**Published:** 2023-12-25

**Authors:** Camila Benmessaoud, Kaylen J Pfisterer, Anjelica De Leon, Ashish Saragadam, Noor El-Dassouki, Karen G M Young, Raima Lohani, Ting Xiong, Quynh Pham

**Affiliations:** 1 Centre for Digital Therapeutics Toronto General Hospital Research Institute University Health Network Toronto, ON Canada; 2 Systems Design Engineering Faculty of Engineering University of Waterloo Waterloo, ON Canada; 3 Healthcare Human Factors Toronto General Hospital Research Institute University Health Network Toronto, ON Canada; 4 Faculty of Media and Arts Humber College Toronto, ON Canada; 5 School of Public Health Sciences Faculty of Health University of Waterloo Waterloo, ON Canada; 6 Institute of Health Policy, Management and Evaluation Dalla Lana School of Public Health University of Toronto Toronto, ON Canada; 7 Telfer School of Management University of Ottawa Ottawa, ON Canada

**Keywords:** digital therapeutics, disease management, heart failure, informal caregivers, mHealth, mobile health, shared care, telemedicine

## Abstract

**Background:**

The COVID-19 pandemic forced the spread of digital health tools to address limited clinical resources for chronic health management. It also illuminated a population of older patients requiring an informal caregiver (IC) to access this care due to accessibility, technological literacy, or English proficiency concerns. For patients with heart failure (HF), this rapid transition exacerbated the demand on ICs and pushed Canadians toward a dyadic care model where patients and ICs comanage care. Our previous work identified an opportunity to improve this dyadic HF experience through a shared model of dyadic digital health. We call this alternative model of care “Caretown for Medly,” which empowers ICs to concurrently expand patients’ self-care abilities while acknowledging ICs’ eagerness to provide greater support.

**Objective:**

We present the systematic design and development of the Caretown for Medly dyadic management module. While HF is the outlined use case, we outline our design methodology and report on 6 core disease-invariant features applied to dyadic shared care for HF management. This work lays the foundation for future usability assessments of Caretown for Medly.

**Methods:**

We conducted a qualitative, human-centered design study based on 25 semistructured interviews with self-identified ICs of loved ones living with HF. Interviews underwent thematic content analysis by 2 coders independently for themes derived deductively (eg, based on the interview guide) and inductively refined. To build the Caretown for Medly model, we (1) leveraged the Knowledge to Action (KTA) framework to translate knowledge into action and (2) borrowed Google Sprint’s ability to quickly “solve big problems and test new ideas,” which has been effective in the medical and digital health spaces. Specifically, we blended these 2 concepts into a new framework called the “KTA Sprint.”

**Results:**

We identified 6 core disease-invariant features to support ICs in care dyads to provide more effective care while capitalizing on dyadic care’s synergistic benefits. Features were designed for customizability to suit the patient’s condition, informed by stakeholder analysis, corroborated with literature, and vetted through user needs assessments. These features include (1) live reports to enhance data sharing and facilitate appropriate IC support, (2) care cards to enhance guidance on the caregiving role, (3) direct messaging to dissolve the disconnect across the circle of care, (4) medication wallet to improve guidance on managing complex medication regimens, (5) medical events timeline to improve and consolidate management and organization, and (6) caregiver resources to provide disease-specific education and support their self-care.

**Conclusions:**

These disease-invariant features were designed to address ICs’ needs in supporting their care partner. We anticipate that the implementation of these features will empower a shared model of care for chronic disease management through digital health and will improve outcomes for care dyads.

## Introduction

The COVID-19 pandemic forced the spread of digital health to address limited clinical resources for managing chronic health conditions. It also illuminated the population of older patients who could not access this care without an informal caregiver (IC) due to accessibility, technological literacy, or English proficiency concerns, thereby widening the digital divide [1,2]. For example, as heart failure (HF) prevalence increases with age and continues to impose chronic deterioration, individuals living with this disease often experience loss of independence [3,4] and significant declines in their quality of life. As a result, ICs often step in and take on the task of supporting their loved ones with activities of daily living, psychosocial aid, improving and maintaining self-care, and navigating the health care system [5]. There are 2.7 million ICs aged older than 45 years who provide the majority (~75%) of home care services in Canada [6,7]. The rapid transition to digital health further exacerbated the demand on ICs and pushed Canadians toward a dyadic care model in the management of chronic disease, where patients and ICs work to manage care together [8].

Traditional, in-person care for patients with HF consists of infrequent specialist appointments, medication, surgery, and device therapies [9]. As a result, care tends to be more passive, waiting on a decompensation or hospital visit as the impetus to make an adjustment to the patient’s care plan. However, with the proper tools and resources, self-management can become possible and effective. For example, Medly, a digital therapeutic for HF management, provides users with self-care feedback messages in response to patient-reported physiological measures and symptoms and offers daily remote nurse monitoring. The nurse-led Medly program is the standard of care for HF at the Peter Munk Cardiac Centre and has increased HF-related quality of life, improved health outcomes, and reduced HF-related hospitalizations by 50% [10].

Others have created digital therapeutics with similar impact, including Huma for HF [11], Livongo Health for diabetes [12], and Vinehealth for cancer [13,14]. However, for Medly and others [10-14], the challenge remains that these interventions were designed for patient self-management, missing the opportunity to capitalize on the synergistic benefits of dyadic care [15]. Our previous work identified an opportunity to improve the dyadic experience through a shared model of dyadic digital health, expanding beyond individual HF self-management to include support for ICs [2]. The core concept is that this alternative model of shared dyadic care can be added as a module to Medly. We refer to this module as “Caretown.” Through “Caretown for Medly,” ICs concurrently expand the patient’s ability for self-care while acknowledging their own personal needs to facilitate a greater level of support.

In this study, we extend our previous work [2] and outline the output of this qualitative, human-centered design study. Here, we outline our systematic design methodology for Caretown and report on the output (6 core features) of this methodology applied within the space of ICs’ dyadic shared care of HF management.

## Methods

### Overview

Our previous work outlines the underlying data collection and analysis of a qualitative descriptive study comprised of interviews with ICs who have lived experience supporting individuals with HF in Ontario, Canada [2]. However, while Medly is used as a benchmark technology to apply this new model of dyadic care for ICs supporting their loved ones living with HF, Caretown for Medly was intentionally co-designed for adaptability to support any chronic disease self-management tool. Our co-design process included the following: needs assessment; framework development; and requirements, design, and feature validation.

### Needs Assessment

To ensure data saturation, we conducted a needs assessment informed by a convenience sample of 25 IC interviews enrolled in the Medly HF management program at the University Health Network. These included 5 additional interviews beyond those reported elsewhere [2]. The ICs supporting patients on the Medly program were invited to be research partners in the co-design of Caretown. A semistructured interview guide directed the 25 IC remote interviews conducted by NED (woman, MSc; research associate) and CB (woman, MHI; research analyst) without an established relationship with participants before study commencement. Audio-recorded interviews were conducted either through telephone calls or video calls using Microsoft Teams (Microsoft Corporation) based on participant preference and lasted approximately 1 hour in duration. These interviews explored ICs’ personal goals and the barriers they faced in achieving them through the following three main themes: (1) ICs’ relationship with their care recipients and their experiences with caregiving, (2) the IC’s role in and views on the Medly program, and (3) opportunities to improve the Medly experience to further support the dyad [2]. Analysis of interviews was conducted using NVivo (QSR International) software through the preliminary development of a codebook based on IC activities outlined by Buck et al [16] and a review of the inherent initial themes identified in the first 6 transcripts. Interviews were analyzed by NED, CB, KGMY (woman, MSc student), RL (woman, MHI; research associate), and QP (woman, PhD; scientific director) to discuss key ideas, thoughts, and potential feature suggestions, all with formal training in qualitative research methods. A final version of the codebook for formal data analysis was developed iteratively after establishing consensus in the codes with input from 4 coders. Each interview underwent thematic content analysis [17] independently by 2 coders, with themes both derived deductively (eg, based on the interview guide) and inductively refined to incorporate additional identified themes [17]. To ensure the quality of this study, we looked at the 8 big-tent criteria for high-quality qualitative research [18]. Methodological rigor was sought using relevant frameworks. The context was preserved through rich descriptions of the sample. We used theoretically informed data collection and analytical methods. Our reflexivity and positionality addressed the additional key criterion of sincerity to be transparent to ourselves and our readers, aware of our motivations for pursuing this work and any biases we may have held in the process of data collection and analysis. Additional details are available elsewhere [2].

### Framework Development

**Figure 1:**
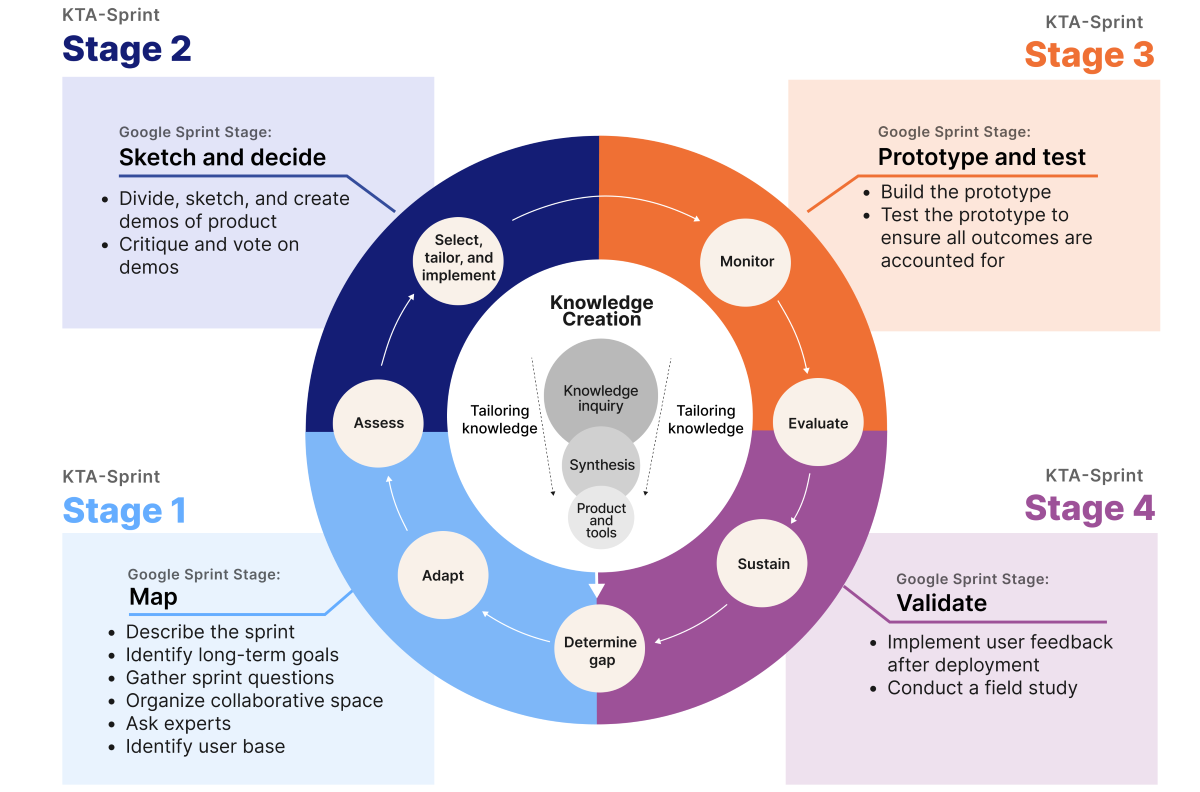
The Knowledge to Action (KTA) Sprint provides the infrastructure for quick and systematic iteration of user-directed solution concepts through 4 stages. The KTA Sprint fuses the KTA framework with Google Sprint methodology for conducting early rapid iterative evaluation positioned for subsequent piloting and rigorous pragmatic evaluation.

KJP and CB developed an adaptation of an overarching framework positioned by an informal scoping review focusing on disease-invariant evidence to support ICs’ unmet needs for chronic disease comanagement. Our team wanted a framework that is clinically relevant, positioned well for translational research, and supportive of a nimble, agile research environment to avoid the 17-year lag [19] between research and translation. To address this, (1) we looked to the Knowledge to Action (KTA) framework [20] for its ability to translate knowledge into action, and (2) we borrowed the Google Sprint [21] from industry titans to quickly “solve big problems and test new ideas,” which has been effective in the medical and digital health spaces [22-24]. Specifically, we blended these 2 concepts into a new framework we call the “KTA Sprint.” The KTA Sprint merges user-centered and participatory design [25,26] with rapid prototyping methods [21,27] to provide an actionable framework (Figure 1). The result is the infrastructure for quick and systematic iteration of user-directed solution concepts through 4 stages. Stage 1 identifies long-term goals, assesses needs, and establishes a user base. This first stage aligns the “determine gap,” “adapt,” and “assess” aspects of the KTA cycle [20] with the “map” process of the Google Sprint [21]. Stage 2 commences solution thinking, where concepts are sketched and critiqued, and the most promising ideas are voted on. This second stage aligns the “assess” and “select, tailor, and implement” aspects of the KTA cycle [20] with the “sketch and decide” process of the Google Sprint [21]. Stage 3 runs with these solution sketches to develop a Goldilocks’ quality (ie, “just right” fidelity) prototype to assess and test the workflow. This third stage is similar to the “monitor” and “evaluate” aspects of the KTA cycle [20] and aligns with the “prototype” stage of the Google Sprint [21]. Stage 4 implements user feedback after a pilot deployment to further improve the prototype. This fourth stage aligns with the “evaluate” and “sustain” aspects of the KTA cycle [20] and the “validate” stage of the Google Sprint [21]. This KTA Sprint is well positioned for early conceptualizations, with rapid iterative evaluation conducted early on. While stages 3-4 were outside of the scope of this human-centered study, the focus of this paper is on stages 1 and 2, the design aspects of our collaborative, participatory, iterative design sprint for Caretown.

### Requirements, Design, and Feature Validation

We translated user needs and requirements into designs and features. Human-centered design principles drove our feature validation. The opportunity for the core features described below arose from 25 semistructured IC interviews and was further vetted by a feature prioritization survey with a subset of 11 ICs using a 5-point Likert scale set of responses. Through a standardized approach to product design, we can provide a more tailored experience to concurrently address patient and IC needs [2]. To explore this opportunity within the Caretown for Medly context, we conducted a stakeholder analysis, a market scan, and a user needs assessment to better understand the fundamental caregiving processes and experiences with dyadic HF digital health management. The stakeholder analysis revealed that ICs in care dyads could be classified into 1 of 3 dyadic typologies in the use of digital health tools [28]., which include: IC-oriented (ie, IC as a primary user), collaborative (ie, IC as a secondary user), or patient-oriented dyads (ie, IC as a nonuser). A market scan was conducted to identify existing dyadic chronic disease management programs and digital products to extrapolate the feasibility, effectiveness, and sustainability of potential features along with existing gaps. In reviewing the several IC applications that exist on the market [29], we found that many supported the management of care tasks but lacked disease-specific dyadic symptom management features [15,30]. Here, we explore how existing chronic disease self-management tools can be adapted to support shared care. To identify user needs, a total of 25 research partners who self-identified as informal ICs of an individual living with HF were recruited through convenience sampling. As described elsewhere [2], research partners completed a preinterview demographic questionnaire and a semistructured interview exploring daily IC experiences to support patients with HF and the role of technology in supporting ICs in achieving their caregiving goals.

### Ethical Considerations

All recruitment and data collection activities, including ethics review, informed consent, privacy, and compensation, were approved by the University Health Network Research Ethics Board (REB 20-5238). Compensation was provided based on the Canadian Institutes of Health Research SPOR recommendations [31].

## Results

### Principal Findings

**Figure 2:**
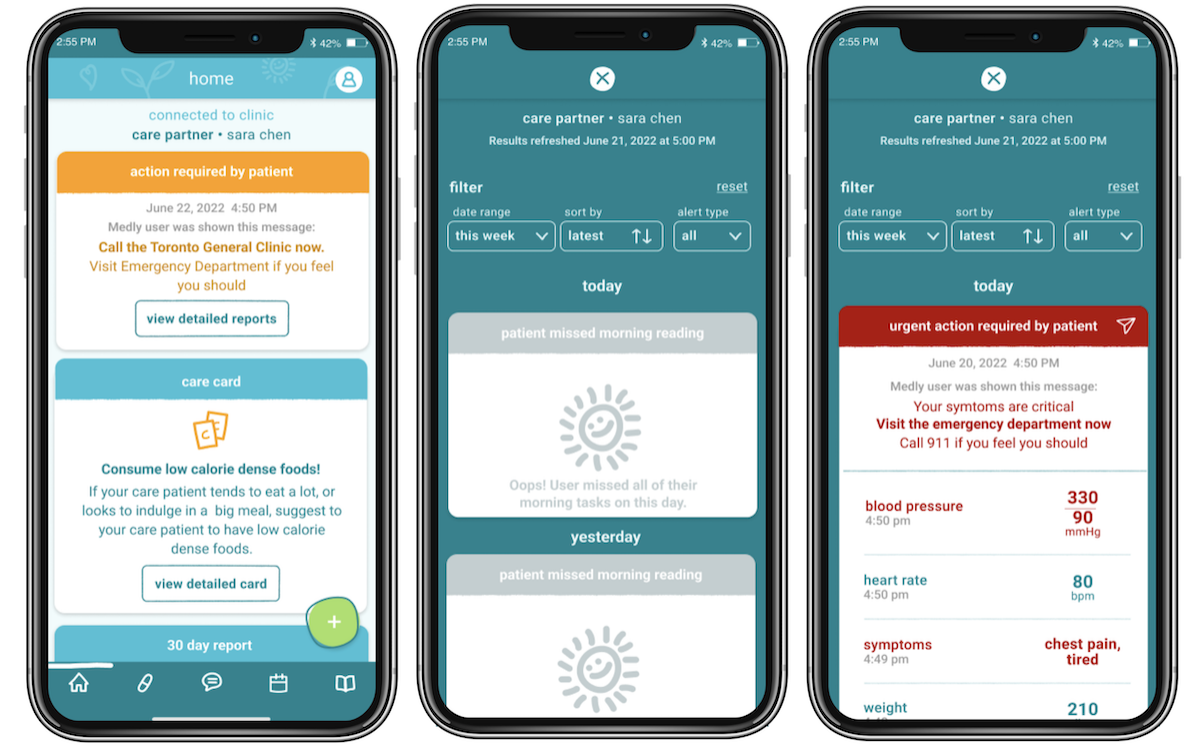
Live reports provide a snapshot of the patient’s health status at the present time through data sharing. They are color-coded according to urgency and contain information about the actions required for symptom management (eg, visiting the emergency department) along with the patient-reported outcome measure (PROM) that triggered the alert. The expanded version provides additional information within the context of the PROM data, highlighting the values that contribute to the level of urgency or remain grayed out for missed readings.

**Figure 3:**
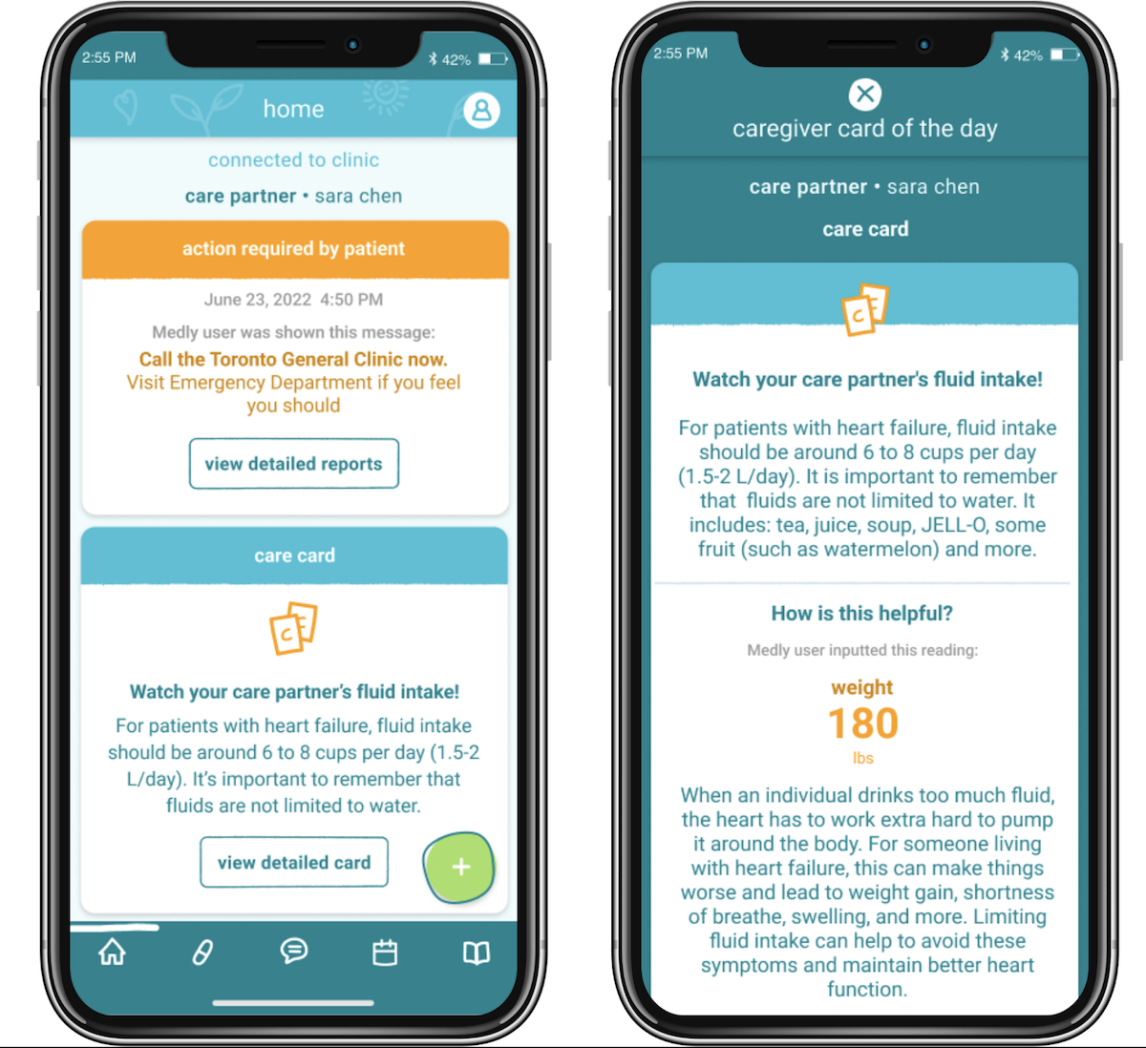
Care cards provide a color-coded tip of the day that matches the current action required on the live report. Upon expanding the care cards, more information is available about which patient-reported outcome measure (PROM) contributed to receiving the tip and can be saved for later reference. If the informal caregiver (IC) wishes to learn more, they can be connected to additional, relevant educational resources.

**Figure 4:**
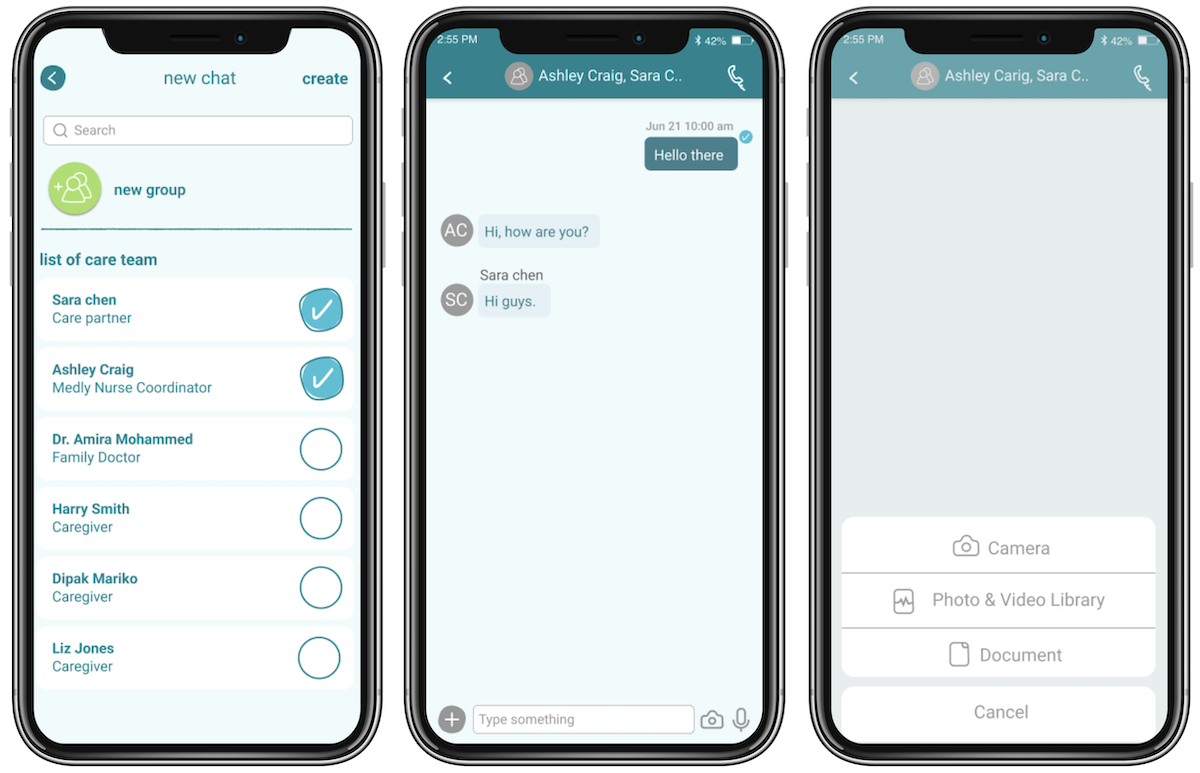
Messaging provides informal caregivers (ICs) with the opportunity to connect with their patients’ clinical team. They can share photos (eg, swollen ankles), videos, documents, and live reports to facilitate communication and receive appropriate feedback to elicit effective care.

**Figure 5:**
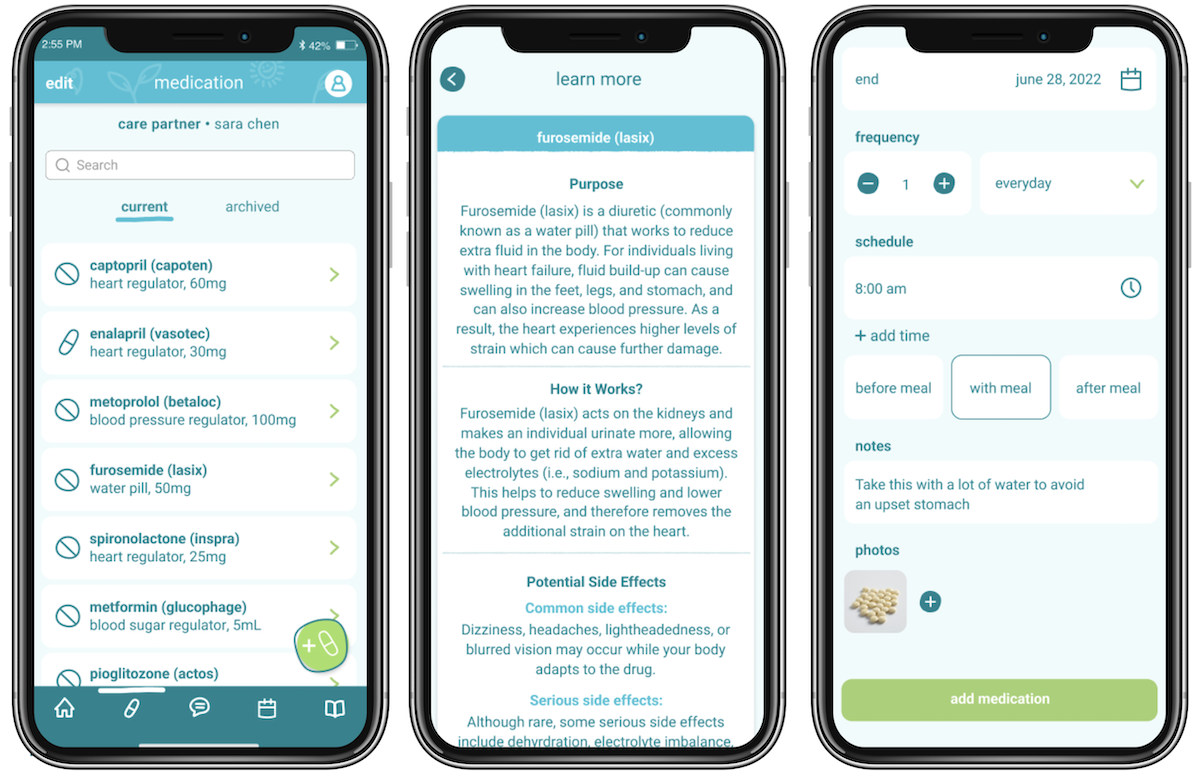
The medication wallet allows informal caregivers (ICs) to input their patients’ medication details. It provides reminders at the defined dosage intervals along with the purpose, dose, frequency, and schedule. The expanded view provides additional information, including photos of the medication packaging and label, along with a free-form notes section where ICs can include details such as adverse reactions. Accessible through the “learn more” button, ICs can seek medication education to learn more about the drug’s purpose, function, and potential side effects.

**Figure 6:**
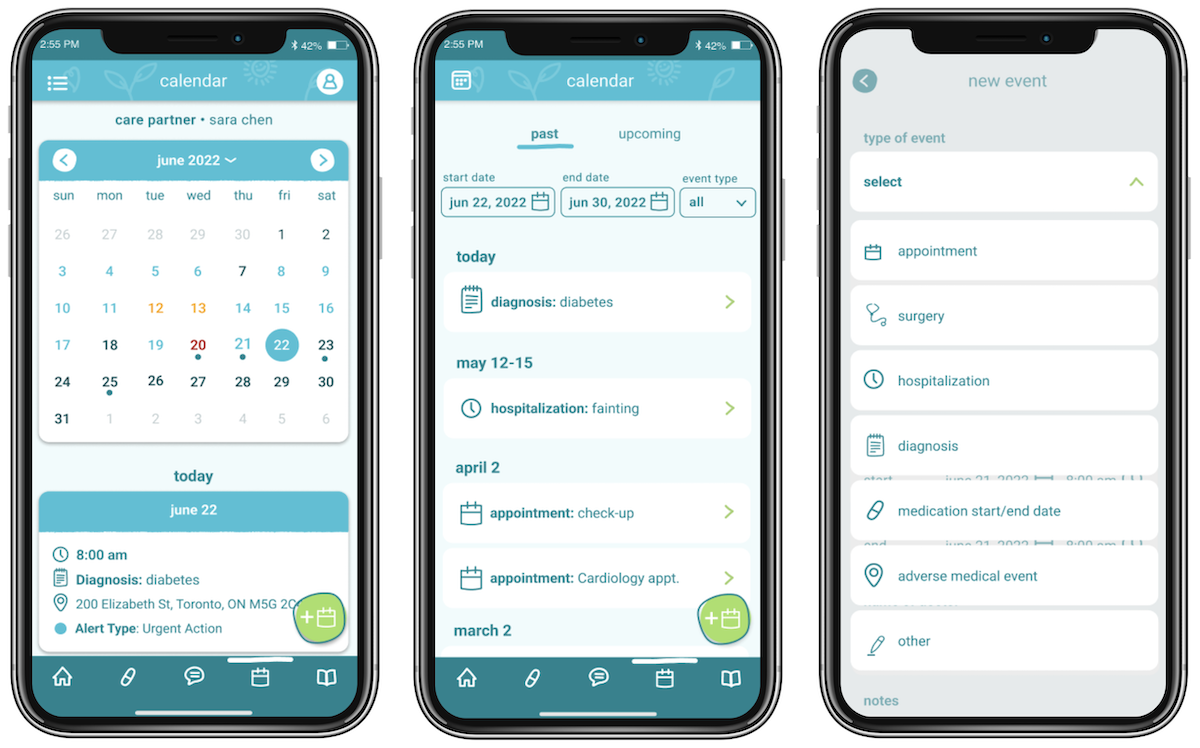
The timeline function allows informal caregivers (ICs) to keep all significant medical events, such as diagnoses, surgery dates, and hospitalizations, in one place. Informed by the live reports, the calendar and summary view dates are also color-coded to reflect the alert type received on the given date. The expanded list view summarizes all events within a specified date range.

**Figure 7:**
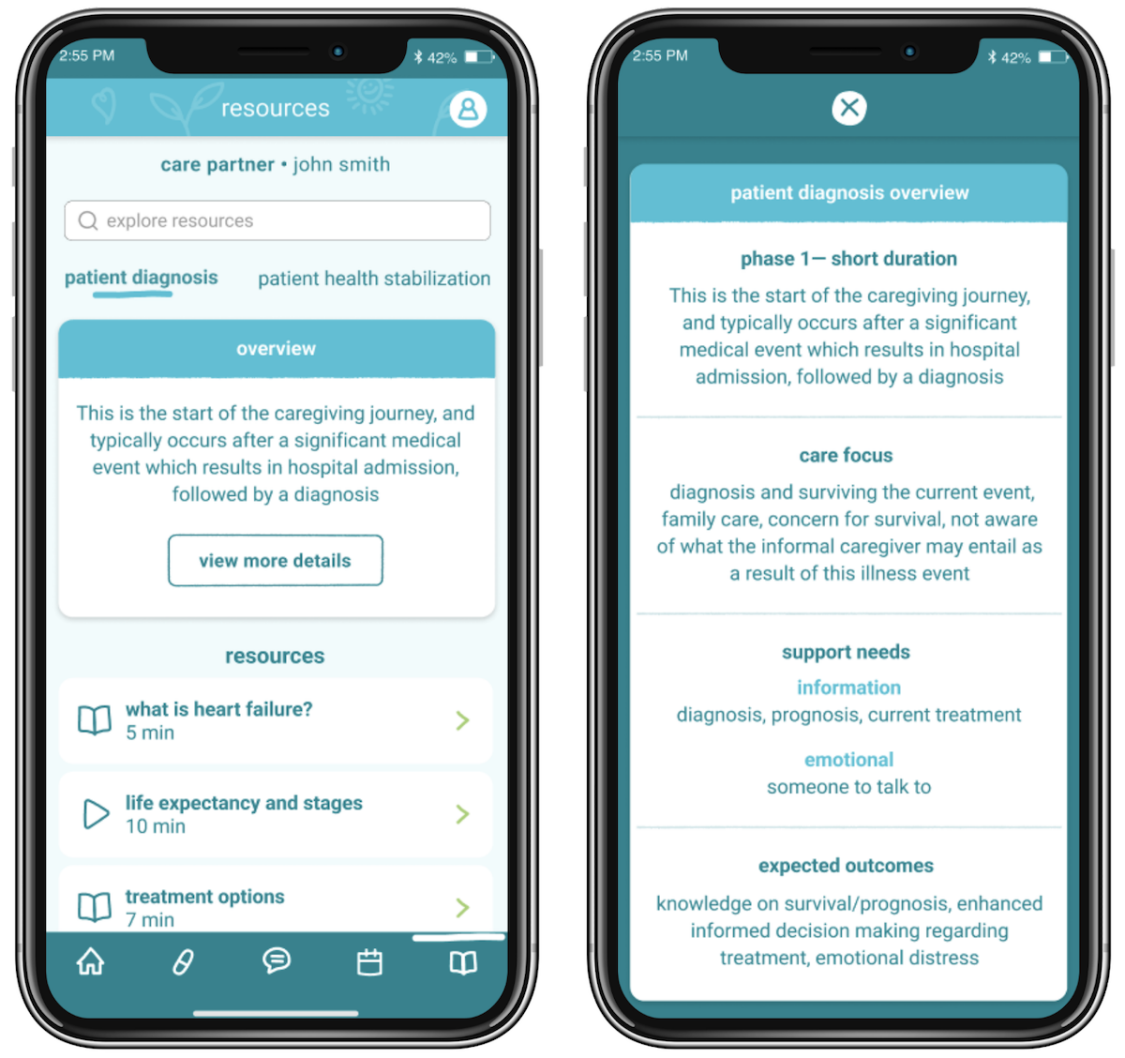
The caregiver resources feature provides informal caregivers (ICs) with timely support resources that are vetted from credible resources. In line with the “timing it right” framework [32], this section is divided into the various stages of caregiving and accompanied by the appropriate resource and support needs. For example, disease-specific educational resources, resources on adjusting to life as an IC, and ICs’ mental and physical health resources would be provided in various forms (academic literature, gray literature, videos, etc).

Based on the user needs assessment, we identified 6 core features pertinent to enhancing dyadic management of a chronic condition, including live reports, care cards, messenger, medication wallet, medical timeline, and caregiver resources (Figures 2-7). Each of these features was further corroborated by the literature and qualitative narrative exposition below. A total of 5 of these 6 features were included in the feature prioritization survey completed by 11 ICs; the live reports feature was previously integrated into Medly.

### Live Reports: Overview and Need

Live reports (Figure 2) are a data-sharing feature that provides a patient’s circle of care with a live view of their health status. ICs will be able to see their patients’ patient-reported outcome measures (PROMs) in real time. Color-coded by urgency, ICs can be notified when a patient’s PROMs indicate that no action is required, action is required, or urgent action is required to support dyadic symptom management and improve ICs’ awareness. Furthermore, to improve adherence, ICs are also notified when a patient misses a daily reading. As evidenced by the participant interviews, caregivers suggested that sharing PROMs may help improve dyadic communication, increase the IC’s understanding of HF and its symptoms, and lead to more proactive care by increasing the IC’s awareness of the patient’s health status.

It would be great for me to be able to jump on the app and just have a look and see, well, how many pillows did she sleep with? Is she having a hard time doing the stairs, that kind of thing.

C24

It might just clarify for me, OK, it’s all right, I can go [out] today. Because otherwise I may go ‘oh, I don't know, I'll just stay here again.’

C14

### Care Cards: Overview and Need

Daily tip cards (Figure 3) can provide prescriptive, actionable, and practical symptom management suggestions to support ICs’ frequent feelings of uncertainty related to daily PROMs. Our interview findings reflect the current state of care, in which ICs attest to receiving inadequate guidance on how to best perform their caregiving role.

It’s clear that I was a caregiver but there wasn’t anyone necessarily looking out for how to keep me in the loop… So there is on one side of this the acknowledgement that you need to have your caregiver present but there wasn’t many tips, guides, supports for caregivers.

C27

I would way rather people be absolutely honest with me, and my performance as a caregiver so I know what to do. I want someone who would be very constructive in their communication to help me be a better caregiver.

C29

### Direct Messaging: Overview and Need

The messaging function (Figure 4) allows for improved communication between the IC and the patient’s professional health care team, providing an avenue for ICs to stay informed and resolve concerns. From our experience with Medly providers, the availability of the health care team plays an integral role in providing reassurance and peace of mind to ICs. We have found that ICs who perceive the health care team to be readily available are better able to cope with uncertain situations.

It's nice to know that I can get a hold of a nurse or I can get a hold of someone if I get a little anxious or I have a question, that means a lot to me.

C26

And the nurse is an email away, like seriously an email away or a phone call away. It’s just – I can’t say enough.

C28

### Medications Wallet: Overview and Need

The medication management wallet (Figure 5) stores all patient medication information in one place. Drug information overload, which is especially common for those ICs supporting patients living with multiple comorbidities, often leaves ICs feeling overwhelmed and uncertain about medication management requirements.

The challenge is because there's so many pills ... do you take all seven at the same time? Are you supposed to space them out between time?

C08

I think that would take stress off of her [patient] and be able to confidently say, “Here’s her most recent list of medications.” Because even when you get a printout from Rexall, it’s just a list of meds. So if there’s a way for her to update that in a clean way that would be really good.

C27

I kind of had a panic, and I thought, is he taking the wrong kind of pill, does it get put in incorrectly. That would be really helpful if you could say, he’s taking this, it's the blood thinner, it's this much he gets a day, and yeah and it's for blood thinning, whatever.

C29

### Medical Timeline: Overview and Need

ICs are often responsible for managing and overseeing 2 schedules: their own and those of their patients. The medical health care timeline (Figure 6) outlines significant medical events like diagnosis dates, surgeries, hospitalizations, and medication changes to facilitate effective appointment conversations and efficient care management.

They always say, when was your first surgery, when was this, when was this?... if it was all on a spreadsheet we wouldn't have to go through that every time we see a new doctor.

C18

Every time he came home from the hospital – I don’t put things on Facebook, but I just would write “home” and post it… And that was it. So I could go back into my posts and say, “oh we came home on this date, we came home on this date, and this date, and this date.” And that was the only way I could figure out when we came home.

C29

### Caregiver Resources: Overview and Need

Caregiver resources (Figure 7) [32] were borne out of our user needs assessment interviews, which highlighted 2 major factors that affect a high-quality and supportive environment for ICs: the issue of IC self-neglect and a lack of resources, which manifests in self-inefficacy. ICs, especially those in IC-oriented dyads, often devote a significant amount of their time and prioritize tending to their patients’ care, causing them to neglect their own needs.

I have dropped off all my self-care…like I said, I was neglecting myself, my own health.

C20

Yes, it’s [capacity to achieve personal goals] very limited. Everything [gets] cut down to size... Really, I have to be at home to make sure he’s OK.

C14

The lack of resources available to support these typically untrained ICs in providing quality care for their patients gives rise to IC self-doubt and a lack of confidence.

I wish at the beginning, particularly before we even were referred to Toronto that we had learned more – we have received more information right at the beginning. And even still, there’s probably more information I need to learn about heart failure that I just don’t know yet and I don’t know what I don’t know, right?

C28

## Discussion

### Overview

Our discussion is organized based on the anticipated effects of each of the 6 core features, with corroboration from existing literature. It concludes with a more action-focused, generalizable resource outlining key components and opportunities to support ICs informed by this study (Table 1).

**Table 1:** Summary of opportunities to support dyadic management of heart failure (HF) and their key components to address unmet dyad needs.

Opportunity	Summary	Solution	Key components
Opportunity to enhance data sharing to foster improved dyadic communication and decisions	Informal caregivers (ICs) would appreciate improved data sharing and a salient overview of the patient’s health status to foster improved dyadic communication and decision-making.	Live reports	Daily records of patient-reported outcome measures (PROMs) Reminders to take missed readings to improve clinical adherence History of previous readings
Opportunity to enhance guidance for the caregiving role	ICs would appreciate actionable and prescriptive feedback for practical dyadic symptom management.	Care cards	Actionable, prescriptive tips, and education based on alert types associated with daily PROMs
Opportunity to dissolve the disconnect across the circle of care	ICs would appreciate a platform to unify care plans and enhance communication with professional health care teams.	Messenger	1:1 messaging with the patient’s nurse Direct group messaging between the patient, IC, and health care team Attachment sharing (photos, videos, live report readings etc)
Opportunity to improve guidance on drug management	ICs would appreciate a medication management system to help track medication details like name, purpose, dosage, and frequency.	Medication wallet	Medications list Purpose for medication Time, dose, and frequency Medication education
Opportunity to improve IC access to a patient's medical history	ICs would appreciate the ability to track patients’ medical histories.	Medical timeline	Self-entry calendar and timeline of significant patient events (eg, diagnosis date, hospital visits, and appointments)
Opportunity to improve IC resources and support their self-care	ICs would appreciate disease-specific education to understand prognosis and supportive strategies across the HF trajectory (early diagnosis through palliative care and end-of-life support).	Caregiver resources	IC mental health and physical health resources Educational resources

### Live Reports to Foster Improved Dyadic Communication and Decisions

Existing work has shown this type of data-sharing feature has the potential to improve quality of life for both the patient and caregiver, along with the quality of patient care, by improving transparency and awareness among the dyad [33], enhancing the accuracy of data measurements [33], providing greater peace of mind [34], and supporting enhanced communications within the dyad, enabling ICs to better develop personal coping strategies [35]. While these improvements reflect the views of our interviewed ICs, it is also important to note that in some cases, data sharing may negatively impact the trust held by patients toward their ICs as they feel an invasion of privacy [36], increase caregiver anxiety associated with concern for the patient’s health, or augment relationship tensions [34]. The value of data sharing must therefore be weighed on a dyad-by-dyad basis. We also noted that this functionality may be of higher interest to ICs who are not already accessing the platform on behalf of the patient or who do not live with the patient.

### Care Cards to Enhance Guidance for the Caregiving Role

Although it is common practice for ICs to receive disease-specific education from nurses upon hospital discharge, this may be insufficient given the extent of care required to support patients and the complex and dynamic patient and IC needs over time [37,38]. Traditionally, IC applications have addressed only part of the ICs’ need for caregiving guidance through untailored (ie, not disease-specific) educational resources or patient support tools [39]. There is high value in providing disease-specific educational resources, as they have been shown to improve disease management, patients’ and ICs’ quality of life [40,41], and the ICs’ confidence and effectiveness in their caregiving role [42].

### Direct Messaging to Dissolve the Disconnect Across the Circle of Care

The literature also supports the direct messaging feature, noting that the majority of care occurs outside a health care facility and that connecting ICs with a nurse has been identified as a helpful support mechanism [39,43]. We expect this feature will mitigate how caregivers have stated they often feel neglected in their needs by the health care team [44], and protect them against feeling lost in their unchosen role without support [45].

### Medication Wallet to Improve Guidance on Drug Management

Specifically, we expect this feature will support ICs by helping them to better understand the purpose of the patient’s medications and empower the IC’s self-efficacy in comanaging this task. Managing HF, like many other chronic conditions, often involves managing polypharmacy. Consistent with research [46,47], our qualitative descriptive research revealed the propensity for ICs to support their patients with the administration and management of medications (eg, dosages, timing, and frequency) to improve adherence [48].

### Medical Timeline Tracker to Improve Informal Caregiver’s Access to Patients’ Medical History

ICs carry a substantial mental load to remember appointments, and significant medical events can contribute to caregiver burden. Calendars are commonly used by ICs as an effective organizing tool [49-51], and contribute to positive and improved care coordination [52]. Typically, this is tracked using nondigital methods; however, there is a need for digital health care applications to build solutions to standardize and support information management [53].

### Caregiver Resources to Improve Dyadic Outcomes

Our results were in line with other studies reporting how prioritizing their patients’ care causes ICs to neglect their own mental, emotional, and physical health needs [54,55]. This self-neglect broadly accounts for 7 of the 10 highest-scored unmet IC needs [56]. Providing ICs with tailored education, peer support, and direct communication with the clinical care team (as described above) can help resolve their perturbations. According to the “timing it right” framework, ICs require different types of support and education across the various stages of caregiving in order to facilitate more effective care for the patient while also improving the ICs’ well-being and self-efficacy [32]. Often, there is a lack of disease-specific education pertaining to disease prognosis, how to properly provide care for patients after diagnosis, and how required supports change and shift for palliative care and end-of-life support [5,39,57]. As a developing area of research, there is inconclusive evidence as to which aspects of IC support are most effective in improving overall IC well-being. However, current literature suggests that education combined with peer and professional support can improve mental well-being [58]. While finding the balance of which types and formats of support and resources to provide may be nuanced, our interviews illuminated several candidate components, including linking to trusted sources, the development of maintained resources, IC wellness check-ins to prompt self-care, or creating groups (moderated or unmoderated) for ICs to connect through peer support. Corroborated by research [54], we expect that providing tailored knowledge to educate and support the ICs in times of uncertainty will improve their clinical knowledge and coping skills to reduce their stress and enhance their well-being.

### Actionable Insight Into Opportunities to Support Dyadic Management

Based on feedback from participants, we have amalgamated 6 broad opportunities and how 6 solution components may address these opportunities (Table 1).

### Conclusion

This study outlines the systematic design and development of Caretown for Medly, a new model of dyadic care for ICs supporting their loved ones living with HF. We designed the KTA Sprint to nest within the broader KTA framework. More broadly, we presented 6 core disease-invariant features to support ICs in care dyads to provide more effective care and to capitalize on the synergistic benefits of dyadic care. These 6 features were designed to be customizable to suit the patient’s condition, informed by stakeholder and task analysis, corroborated with the literature, and vetted through user needs assessment interviews. These features include: (1) live reports to enhance data sharing and facilitate appropriate IC support, (2) care cards to enhance guidance on the caregiving role, (3) direct messaging to dissolve the disconnect across the circle of care, (4) medication wallet to improve guidance on managing complex medication regimens, (5) medical events timeline to improve and consolidate management and organization, and (6) caregiver resources to provide disease-specific education and support their self-care. We anticipate that both patient and caregiver outcomes will improve by enabling a dyadic model of digital health care. This model should reflect the shared nature of care and effectively support the holistic needs of this dyad as they collaboratively experience HF.

As our team continues to build the Caretown model, our next steps focus on stage 3 (prototype, test, and monitor) of the KTA Sprint. As part of this stage, we will facilitate usability testing sessions with Medly caregiver partners to test the prototype. Feedback from this stage will be used to refine, evaluate, and validate our design, completing stage 4 of the KTA Sprint cycle.

### Data Availability

The data sets generated and/or analyzed during this study are not publicly available due to sharing having not been part of the informed consent agreement.

## Abbreviations

HF: heart failure
IC: informal caregiver
KTA: Knowledge to Action
PROM: patient-reported outcome measure
